# Reciprocal Sliding Friction Model for an Electro-Deposited Coating and Its Parameter Estimation Using Markov Chain Monte Carlo Method

**DOI:** 10.3390/ma9040237

**Published:** 2016-03-25

**Authors:** Kyungmok Kim, Jaewook Lee

**Affiliations:** School of Aerospace and Mechanical Engineering, Korea Aerospace University, 76 Hanggongdaehang-ro, Deogyang-gu, Goyang-si, Gyeonggi-do 412-791, Korea; kkim@kau.ac.kr

**Keywords:** sliding, friction, electro-deposited coating, Markov Chain Monte Carlo method

## Abstract

This paper describes a sliding friction model for an electro-deposited coating. Reciprocating sliding tests using ball-on-flat plate test apparatus are performed to determine an evolution of the kinetic friction coefficient. The evolution of the friction coefficient is classified into the initial running-in period, steady-state sliding, and transition to higher friction. The friction coefficient during the initial running-in period and steady-state sliding is expressed as a simple linear function. The friction coefficient in the transition to higher friction is described with a mathematical model derived from Kachanov-type damage law. The model parameters are then estimated using the Markov Chain Monte Carlo (MCMC) approach. It is identified that estimated friction coefficients obtained by MCMC approach are in good agreement with measured ones.

## 1. Introduction

The kinetic friction coefficient is one of informative quantities describing contact degradation between two bodies. Sliding tests are typically conducted for determining the kinetic friction coefficient. The conventional friction coefficient evolution of a low-friction coating can be divided into three stages: the initial running-in period, a steady-state sliding, and the transition to higher friction [1,2,3]. The friction coefficient remains steady, following the initial running-in period. During a steady-state sliding, the thickness of a low-friction coating is progressively reduced. After a steady-state sliding, the friction coefficient increases rapidly. The transition from steady-state to a higher friction state is attributed to the appearance of the substrate at the contact surface [1]. The growth rate of the friction coefficient in the transition was described with Kachanov-type damage law [2,3]. It was identified that two parameters can describe the growth rate of the friction coefficient resulting from the increase of substrate size appeared at contact. A fretting model based on Kachanov-type damage law was proposed for describing a friction coefficient change in the transition [4]. The fretting model was found to adequately describe the friction coefficient evolution between an electro-deposited coating between a ceramic ball. However, parameters were not estimated. For the practical use of the model, the model needs to be extended to reciprocal sliding. In addition, parameters in the model should be estimated with an appropriate method.

During a sliding test, it is necessary to identify a slip regime, since contact damage varies with a relative displacement between two bodies [5,6]. Parameters such as slip ratio, slip index, and energy ratio were proposed to identify the transition between slip regimes [7,8]. Slip ratio was defined as actual sliding distance divided by total displacement. Slip ratios of 0.26 and 0.95 indicated the transition between partial and gross slip regimes, and the transition between gross and sliding regimes, respectively. Differently from slip ratio, slip index was determined with stiffness and normal force, and a displacement. A slip index of 10 was found to be at the transition between gross and reciprocal sliding regimes [7,8]. An energy ratio, defined as dissipated energy divided by total energy in a fretting loop, was proposed for criterion of the transition between partial and gross slip regimes.

In this study, reciprocal sliding tests with electro-deposited coatings were conducted using ball-on-flat apparatus. The kinetic friction coefficient was measured, along with the relative displacement between a ball and a flat specimen. The evolution of the kinetic friction coefficient is described with an appropriate mathematical model. Parameters of the mathematical model are then estimated using the Markov Chain Monte Carlo (MCMC) approach [9,10]. The MCMC approach quantifies the magnitude of uncertainties in the estimated parameters based on Bayesian inference [11]. As the estimation result, the MCMC approach provides the Probability Density Function (PDF) of the parameters in contrast to the least square method providing a single deterministic value. From the estimated PDF, the predictive interval (*i.e.*, the range in which future observations will be located) is calculated, and then the reliability of the estimated parameters is identified. The estimated model kinetic friction coefficients are compared with the measured coefficients to validate that the proposed mathematical model describes the evolution of friction coefficient evolution for an electro-deposited coating.

## 2. Experimental Set-Up and Materials

**Figure 1:**
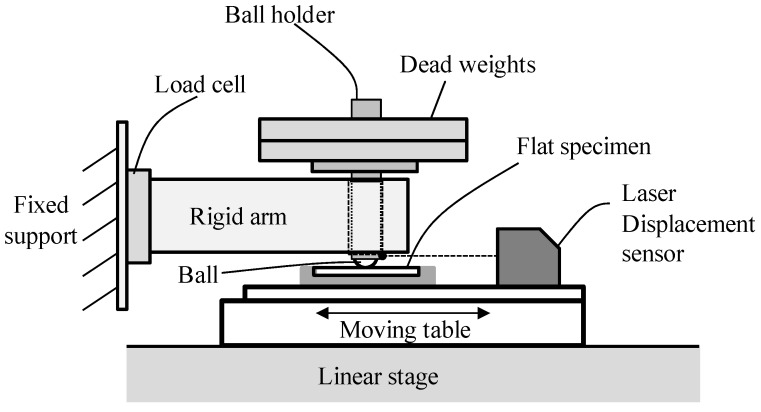
Reciprocal sliding testing machine using ball-on-flat test apparatus.

Sliding tests were conducted with the ball-on-flat plate test apparatus as shown in Figure 1 [12]. One ball and one flat specimen were used for a single test. A ball was fixed in a ball holder connected to a cantilever-type rigid arm. A flat specimen attached on a carriage of a linear stage linearly moved relative to a fixed ball. A laser displacement sensor (LK-081, Keyence Corp., Itasca, Illinois, USA) fixed on a carriage of a linear stage measured the displacement between a ball holder and a carriage. Normal force at contact was induced by dead weights placed on a ball holder. A tangential force at contact was measured with a load-cell connected to the cantilever-type arm. The tangential force and the displacement were recorded on the Labview program.

A ϕ 5 mm ball made of AISI 52100 steel was used. An electro-deposited coating was applied onto the substrate of a cold-rolled high strength steel specimen with an initial thickness of 0.02–0.04 mm. The coating contains an epoxy resin with a metal catalyst and with a crosslinker of blocked aromatic isocyanates. The conditions of electro-deposition were provided in Appendix A.

In this study, normal force similar to those found in automotive seat slide tracks was considered; that is, normal forces of 25 N and 50 N were selected considering the automobile application. A frequency and the imposed displacement between a ball and a flat specimen were defined as 1 Hz and 1 mm, respectively.

## 3. Experimental Results and Interpretation

**Figure 2:**
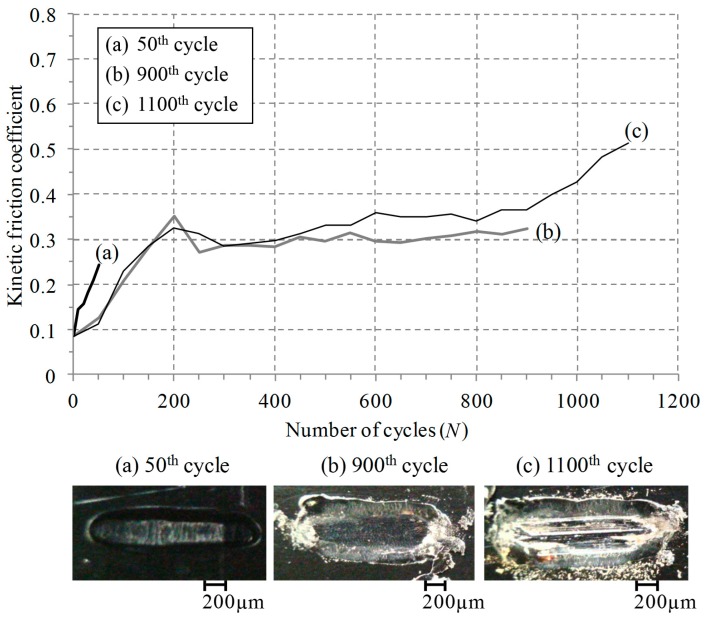
The friction coefficient evolution of an electro-deposited coating at a normal force of 25 N, a displacement of 1 mm, and a frequency of 1 Hz.

A reciprocal sliding test was conducted at a normal force of 25 N, a displacement of 1 mm and a frequency of 1 Hz. In order to observe contact damage at various numbers of cycles, tests were interrupted at the 50th cycle, the 900th cycle and the 1100th cycle, respectively. Figure 2 shows the friction coefficient evolution of an electro-deposited coating. The initial friction coefficient was about 0.1. After 50 cycles (during the initial running-in period), the friction coefficient became 0.25. The worn image captured after 50 cycles shows that a coating layer remained at the contact surface. In a range of 200 to 900 cycles, the friction coefficient remained steady or increased slightly (a steady-state sliding). At the 800th cycle, the friction coefficient started to increase. It is identified from the worn surface at the 900th cycle that the substrate appeared near both contact edges. The image provides why the friction coefficient increased after a steady-state sliding. After the 1100th cycle, the friction coefficient exceeded 0.5. The worn surface image shows that a coating wore off and rough surface appeared resulting from trapping.

**Figure 3:**
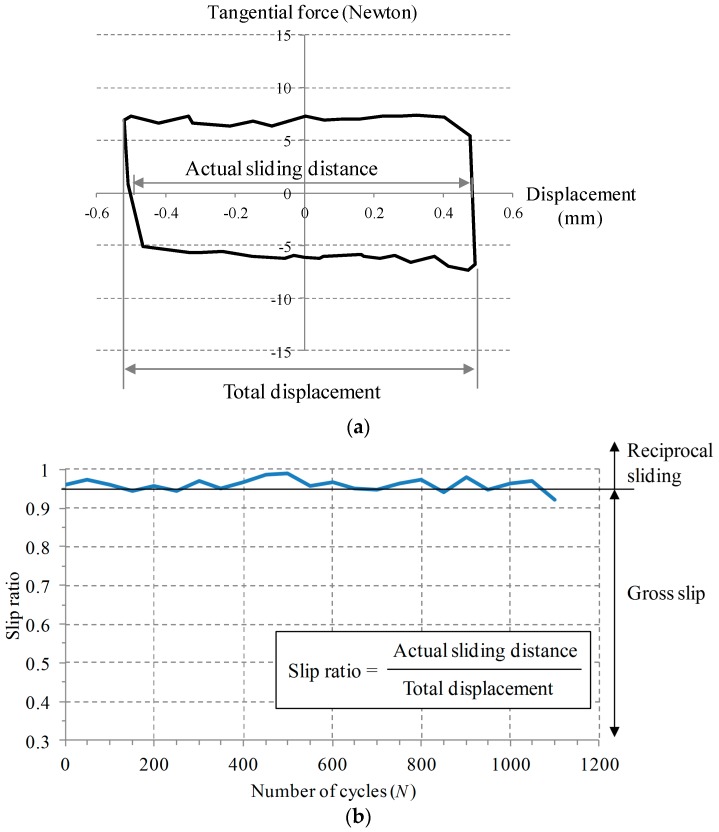
Tangential force *versus* displacement loop (**a**) and slip ratio (**b**) at a normal force of 25 N, a displacement of 1 mm, and a frequency of 1 Hz.

Figure 3 shows slip ratio during a test interrupted at the 1100th cycle. Slip ratio was defined as actual sliding distance divided by total displacement presented in Figure 3a. Slip ratio is useful for identifying a slip regime [8]; slip ratio was found to be 0.26 at the transition between a partial slip regime and a gross slip regime. A slip ratio of 0.95 indicates the transition between a gross slip regime and a reciprocal sliding regime. It was identified from Figure 3b that a test was conducted within a reciprocal sliding regime, since most slip ratio remained above 0.95.

It is necessary to describe the friction coefficient evolution with an appropriate mathematical model. In this study, the transition from the running-in period to a steady-state period was determined with the change rate of the friction coefficient. A linear function is used as the model for the running-in period and for a steady-state sliding. To estimate the parameters of linear functions, the Markov Chain Monte Carlo (MCMC) approach [9,10] was applied. The MCMC approach estimates the posterior Probability Density Function (PDF) of the parameters **θ** conditional on the measured data y. This posterior PDF *P*(**θ**|y) can be calculated based on the Bayes’ rule:

(1) P(θ|y)∝L(y|θ)p(θ)

where *L*(**y**|**θ**) is the joint likelihood function of the data **y** conditional on **θ**, and *p*(**θ**) is the prior distribution of **θ**. In this work, the data **y** is the vector of measured friction coefficients, and the parameter **θ** is the set of parameters of a mathematical function. The prior distribution *p*(**θ**) is set as the uniform distribution. The joint likelihood function *L*(**y**|**θ**) is the multiplication of the likelihood functions for *n* measured data:

(2) $$L\left(y|\theta \right)=\overset{n}{\underset{k=1}{\prod }}L\left(y_{k}|\theta \right)=L\left(y_{1}|\theta \right)\times L\left(y_{2}|\theta \right)\times \ldots \times L\left(y_{n}|\theta \right)$$

The likelihood function for *k*th data (*i.e.*, *L*(*y*_k_|**θ**)) is defined based on the assumption that the error of the measured data against the model follows the normal distribution:

(3) $$L\left(y_{k}|\theta \right)=\frac{1}{\sigma \sqrt{2\pi }}\text{exp}\left\{-\frac{1}{2}\frac{{\left(y_{k}-y^{k}\left(\theta \right)\right)}^{2}}{\sigma^{2}}\right\}$$

where, *σ* is the standard deviation and *y^k^*(**θ**) is the model value corresponding to measured data *y_k_*.

To evaluate the posterior PDF *P*(**θ**|**y**), the MCMC approach is the one of effective sampling methods based on modern computational statistics. Among various sampling method for the MCMC approach, the Metropolis-Hastings (M-H) algorithm, which is the most representative method is applied in this work [9]. Please refer to References [9,10] for the detailed process for M-H algorithm in the MCMC approach.

The parameters of 1100 cycle measured data (Figure 1c) are estimated using the MCMC approach. The mathematical models and their estimation results were summarized in Table 1. The estimated median with 95% upper and lower predictive intervals was provided for each model parameter. Here, the 95% predictive interval means the range in which future observations will locate with a 95% probability. In contrast to the least square method providing a single deterministic value, the MCMC approach estimates the ranges of parameters, which enables us to check the reliability of the estimated parameters; the wider predictive intervals means that the uncertainty of the estimation result is severe, and vice versa.

After a steady-state sliding, the change of the friction coefficient was studied in the literature [2]. That is, the change rate of the friction coefficient was successfully described with a form of Kachanov-type damage law. The relation between the friction coefficient (µ) and the number of cycles (*N*) was obtained as:

(4) $$\frac{d\mu }{dN}=C\times \mu^{n}$$

where, *C* is the friction coefficient rate constant and n is the friction coefficient exponent.

Some studies were conducted for the correlation between the parameters and experimental conditions under fretting and sliding conditions [2,3,13]; the friction coefficient exponent (*n*) was associated with materials of contacting bodies, whereas the friction coefficient rate constant (*C*) was related to surface treatment, normal force and initial coating thickness on coated systems.

If the friction coefficient exponent (*n*) is unity, the friction coefficient is expressed as an exponential function of a cycle. In some cases, the friction coefficient exponent is not equal to unity. If the friction coefficient exponent is not equal to unity, the friction coefficient (µ) is given as:

(5) $$\mu (N)={\left[C\cdot (1-n)(N)+C^{*}\right]}^{\frac{1}{1-n}}\;$$

where, $C^{*}=\mu_{1}^{1-n}-C\cdot (1-n)\cdot N_{1}\;$, μ_1_ and *N*_1_ are a steady state friction coefficient and number of cycles at the end of a steady-state sliding, respectively.

The parameters *C* and *n* in Equation (5) are estimated for the 1100 cycle measured data (Figure 1c). For the estimation, the MCMC approach is again applied. The mathematical model value *y^k^*(**θ**) in Equation (3) is calculated using the derived friction coefficient expressed as Equation (5). The estimation result was provided in Table 2. The friction coefficient exponent (*n*) of the coating was estimated as 3.083 (median) at a normal force of 25 N, a displacement of 1 mm, and a frequency of 1 Hz. The range of the 95% predictive interval for the friction coefficient rate constant (*C*) is wider than that of *n*, which means that uncertainty of the *C* is relatively significant.

**Figure 4:**
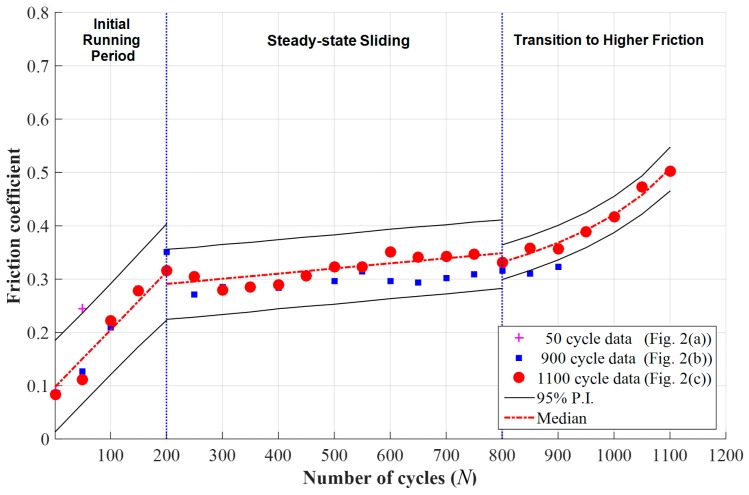
Estimated median and 95% predictive intervals (P.I.) based on the measured data at a normal force of 25 N, a displacement of 1 mm, and a frequency of 1 Hz.

Using the estimated parameters provided in Table 1 and Table 2, the median and 95% predictive intervals of the friction coefficient were calculated and plotted in Figure 4. In addition to 1100 cycle data, 50 and 900 cycle data are marked in the figure. From the comparison of 1100 cycle data and median, it is validated that the derived Equation (5) can successfully fit the friction coefficient for the transition to higher friction region.

**Figure 5:**
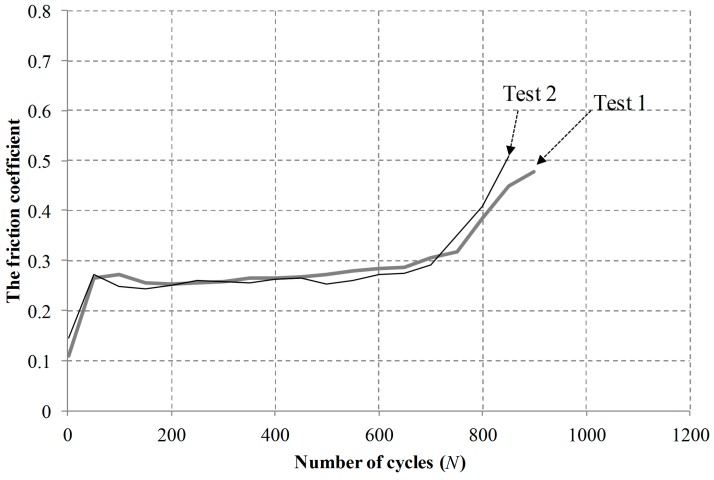
Measured friction coefficient evolutions at a normal force of 50 N, an imposed displacement of 1 mm, and a frequency of 1 Hz.

**Figure 6:**
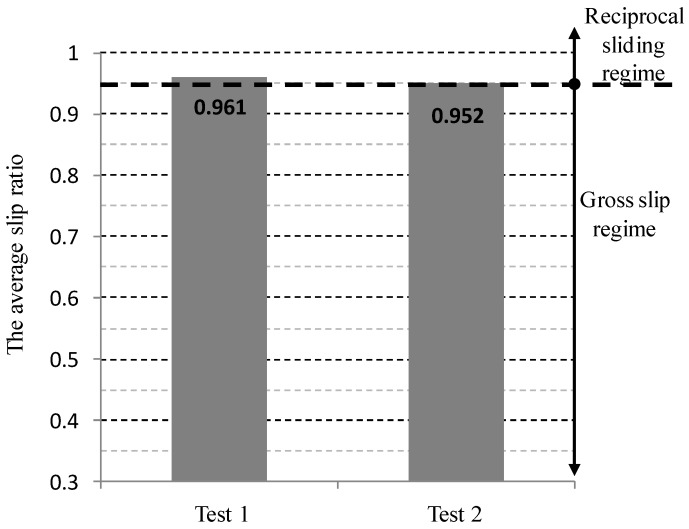
The average slip ratio of tests at a normal force of 50 N, an imposed displacement of 1 mm, and a frequency of 1 Hz.

Additional sliding tests were conducted at a normal force of 50 N, a displacement of 1 mm, and a frequency of 1 Hz. Tests were interrupted at a friction coefficient of 0.5. Two tests were conducted at the same condition. Figure 5 shows friction coefficient evolutions at a displacement of 1.0 mm. Figure 6 shows the average slip ratios of two tests at a normal force of 50 N, and a displacement of 1 mm and a frequency of 1 Hz. The average slip ratios in two tests remained above 0.95, indicating that the tests were completed within a reciprocal sliding regime.

Friction coefficient data presented in Figure 5 were described with an appropriate mathematical model: linear functions for an initial running-in period and for a steady-state sliding, and Equation (5) for the transition to higher friction. The parameters of the mathematical models were estimated for each data using the MCMC approach again, and the estimation results were summarized in Table 3.

**Figure 7:**
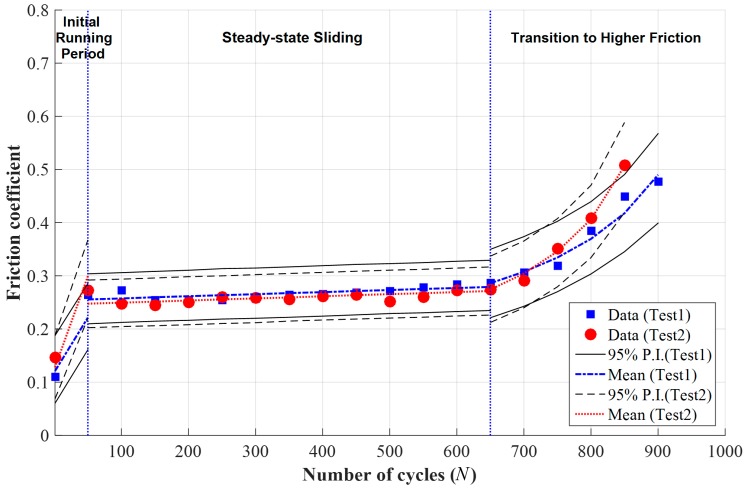
Estimated median and 95% predictive intervals (P.I.) based on the measured data at a normal force of 50 N, a displacement of 1 mm, and a frequency of 1 Hz.

Using the estimated parameters, the median and 95% predictive intervals were calculated and provided in Figure 7. Both test data were successfully fitted with the estimated median, and the most of test data 2 was located inside the 95% predictive interval estimated based on test data 1, and vice versa. It was found that estimated median values for test 1 were close to those for test 2 in all stages. Note again that the 95% predictive interval means the range in which future observations will locate with a 95% probability. This result validates that the derived Equation (5) can successfully describe the friction coefficient for the transition to higher friction region.

In this study, the transition from the running-in period to a steady-state sliding was identified with the change rate of the friction coefficient. A linear function was used for describing the change of the friction coefficient during the running-in period and during a steady-state sliding. In order to identify accurate transition from the running-in periods to a steady-state sliding, it is necessary to conduct further tests and determine the transition statistically. In addition, a non-linear function might be taken into account for description of the friction coefficient during the running-in period.

## 4. Conclusions

The following conclusions were drawn.

- The kinetic friction coefficient evolution between an electro-deposited coating and AISI 52100 steel ball maintained an initial running-in stage, a steady-state sliding stage, and a transition stage to higher friction. In each stage, the change rate of the friction coefficient varied with respect to number of cycles. It was possible to describe the friction coefficient during the running-in period and a steady-state sliding with a linear function. Meanwhile, the friction coefficient in the transition stage was found to be described with the friction coefficient exponent and the friction coefficient rate constant.
- The Markov Chain Monte Carlo (MCMC) method is applied to estimate the parameters of the friction coefficient models. The estimation results validate that the proposed model describes the friction coefficient evolution for an electro-deposited coating adequately. Further work needs to include fretting tests with other test conditions and ball materials (e.g., ceramic balls).

## Figures and Tables

**Table 1:** Mathematical model and its estimated parameters for description of the initial running-in period and the steady-state sliding (Case of 25 N normal force with 1 mm displacement and 1 Hz frequency).

Initial Running-in Period μ = *a*_0_ × *N* + μ_0_			Steady-State Sliding μ = *a*_1_ × *N* + μ_1_	
	*a*_0_	μ_0_	*a*_1_	μ_1_
95% Lower Bound	0.810 × 10^−3^	0.078	7.964 × 10^−5^	0.251
Median	1.080 × 10^−3^	0.102	9.570 × 10^−5^	0.271
95% Upper Bound	1.280 × 10^−3^	0.113	11.498 × 10^−5^	0.291

**Table 2:** Mathematical model and its estimated parameters for the higher friction region (Case of 25 N normal force with 1 mm displacement and 1 Hz frequency).

Transition to Higher Friction $\mu (N)={\left[C\cdot (1-n)(N)+C^{*}\right]}^{\frac{1}{1-n}}\;$ where $C^{*}=\mu_{1}^{1-n}-C\cdot (1-n)\cdot N_{1}\;$		
	*C*	*n*
95% Lower Bound	5.220 × 10^−3^	2.460
Median	9.312 × 10^−3^	3.083
95% Upper Bound	14.169 × 10^−3^	3.522

**Table 3:** Estimated parameters using the measured data of Test 1, Test 2 for the case of 50 N normal force with 1 mm displacement and 1 Hz frequency.

	Initial Running-in Period		Steady-State Sliding		Transition to Higher Friction	
**Test 1**	*a*_0_	μ_0_	*a*_1_	μ_1_	*C*	*n*
95% Upper Predictive Interval	1.489 × 10^−3^	0.097	3.627 × 10^−5^	0.244	0.466 × 10^−2^	1.855
Mean	1.955 × 10^−3^	0.120	3.962 × 10^−5^	0.254	1.278 × 10^−2^	2.790
95% Lower Predictive Interval	2.965 × 10^−3^	0.137	4.463 × 10^−5^	0.266	2.330 × 10^−2^	3.353
**Test 2**	*a*_0_	μ_0_	*a*_1_	μ_1_	*C*	*n*
95% Upper Predictive Interval	2.735 × 10^−3^	0.111	3.399 × 10^−5^	0.235	0.523 × 10^−2^	1.561
Mean	3.567 × 10^−3^	0.124	4.061 × 10^−5^	0.246	1.818 × 10^−2^	2.756
95% Lower Predictive Interval	4.032 × 10^−3^	0.140	4.585 × 10^−5^	0.255	2.875 × 10^−2^	3.202

## Appendix

**Appendix A:** Conditions of electro-deposition.

Condition	Number
Solid	15%~22%
Pigment binder ratio	0.15
pH	5.9–6.3
Conductivity	120–180 µS/mm
Voltage	260 ± 30 V
Electro-deposition time	About 180 s
